# Hospitalizations for cardiovascular events and risk for all cause and cardiovascular mortality in elderly patients with atrial fibrillation treated with oral anticoagulants: beyond preventing thromboembolism

**DOI:** 10.1186/s12877-025-06733-8

**Published:** 2025-12-09

**Authors:** Matteo Candeloro, Qiaosen Chen, Hanne Ehrlinder, Bruna Gigante

**Affiliations:** 1 Department of Innovative Technologies in Medicine and Dentistry, “G. D’Annunzio” University, Chieti, Italy; 2 Department of Medicine Solna, Division of Cardiology, Karolinska Institutet, Solnavägen 30, Stockholm, 171 64 Sweden; 3 Department of Clinical Sciences, Division of Cardiovascular Medicine, Karolinska Institutet, Danderyd Hospital, Stockholm, Sweden; 4 Department of Cardiology, Karolinska University Hospital, Solnavägen 3, Stockholm, 17164 Sweden

**Keywords:** Atrial fibrillation, Ischemic stroke, Ageing, Anticoagulant treatment, Heart failure

## Abstract

**Background:**

Elderly patients with atrial fibrillation (AF) are at increased risk of death, despite oral anticoagulant (OAC) treatment. We estimated the risk of all cause and cardiovascular (CV) death associated with hospitalizations for cardiovascular events (CVEs).

**Setting:**

Retrospective cohort study.

**Methods:**

OAC treated patients (≥ 75 years) (*n* = 2161) discharged from a Swedish cardiology clinic with AF or atrial flutter (AFL) as main diagnosis between 2010 and 2017, were followed up for 12 months. Hospitalizations for CVEs were recorded from the national patient registry and diagnoses combined in five groups: heart failure (HF); stroke/ transient ischemic attack (TIA)/systemic embolism (SE); acute myocardial infarction and peripheral artery disease; bleeding; and other CVEs. We estimated the risk, expressed as hazard ratio (HR) and 95% confidence interval (CI) for all-cause and CV death in each hospitalization group by time-varying Cox regression.

**Results:**

During 12 months of follow up, 178 patients died and 92 were CV deaths. Overall, 391 (18.5%) patients experienced a total of 490 hospitalizations for CVEs. Hospitalizations for any CVEs associated with increased risk (from 3 to 17 folds) for all-cause mortality. Risk for CV mortality increased in patients hospitalized for HF within 90 days (HR and 95%CI) 33.64 (15.97–70.89), for stroke/TIA/SE 14.73 (7.60-28.58) and for other CVEs 8.98 (4.29–18.78).

**Conclusions:**

Hospitalizations for CVEs in elderly AF/AFL OAC treated patients increased the risk for all cause and CV mortality within 12 months from admission for AF/AFL. Hospitalization for HF bared the highest risk, but the residual stroke/TIA/SE risk was noteworthy.

**Supplementary Information:**

The online version contains supplementary material available at 10.1186/s12877-025-06733-8.

## Introduction

Atrial fibrillation (AF) is a common arrhythmia at advanced age and associates with an increased risk for cardiovascular (CV) mortality and hospitalization [1–4], even in patients treated according to clinical guidelines [5, 6]. Oral anticoagulants (OAC) have contributed to a decreased risk for stroke and systemic embolism (SE) in patients with AF regardless of age [2, 7]. However, risk of bleeding is inherently high in elderly patients treated with OAC and mortality linked to both embolism and bleeding is still burdensome [5, 8–10]. 

As life expectancy has improved over the last decades [11], the number of elderly AF patients is expected to double in the years to come [12]. Therefore, identification of conditions associated with an increased risk of death in elderly patients with AF can contribute to improve their survival and wellbeing.

Medical care and in-hospital management of elderly patients are complex. The co-occurrence of multiple diseases (comorbidity), polypharmacotherapy make AF management a challenging task [13]. The estimate 30-day hospital readmission rate for any cause in patients > 65 years old is estimated to 14.6% [14] and AF patients have a higher risk of being hospitalized for CV (pooled incidence 26.3 vs. 15.7 per 100 person-years) than non-CV causes, with older age playing an important role [3]. Other studies focusing on hospital readmissions in AF patients found HF, stroke, myocardial infarction to be the most frequent causes and all associated with higher mortality risk [15]. Moreover, a study from Menichelli et al. found an incidence rate of 12.9%/year; (95% CI: 12.1%−13.6%) for all-cause hospital readmission in AF subjects [16]. However, data often referred to general AF cohorts, including both anticoagulated and not anticoagulated patients and without a specific interest in elderlies nor the impact that multiple hospital admissions in one patient can have.

Heart failure (HF), acute myocardial infarction (AMI) and venous thromboembolism (VTE) are frequent causes of death in AF patients [8, 17]. Moreover, recent studies have confirmed a bidirectional relation between AMI and AF, as well as HF and AF [18, 19]. This relation, together with comorbidities and the peculiar geriatric clinical conditions that go beyond AF (i.e., geriatric syndromes), weakens the patient’s capacity to cope with external stressors and produce a vicious circle leading to multiple hospital admissions [20]. However, current estimates rarely refer to multiple cardiovascular events (CVEs) occurring in the same patient and hold the observation at the first CVE. This can lead to an underestimation of the real burden that multiple admissions for CVEs can generate, in terms of both higher mortality and healthcare costs.

The aim of this study was to analyze if multiple hospitalizations for CVEs (HF, thromboembolism, atherothrombosis and bleeding) affected the risk for all cause and cardiovascular mortality in a population of elderly and very elderly patients with AF- OAC-treated as per guidelines recommendations.

## Methods

### Study population

The Carrebean-elderly (C-Atrial fibrillation: Risks and Benefits of Anticoagulation in the elderly) study population has previously been described [21]. Briefly, 2943 consecutive patients ≥ 75 years discharged from the Department of Cardiology at Danderyd´s University Hospital (Stockholm, Sweden), with AF or atrial flutter (AFL) as main diagnosis between November 1 st 2010 and December 31 st 2017 were included. All patients have been followed up for incident CVEs and death until December 31 st, 2019. The study was approved by the Regional Board of Ethics in Stockholm (Dnr 2016/63 − 31/1 and Dnr 2017/1520-32). All participants have given their written and informed consent. The study was conducted in accordance with the principles of the Helsinki Declaration.

At inclusion, demographic, anthropometric, clinical and biochemical data were collected, as previously reported [21]. Medical history, CV risk factors as well as concomitant treatment with CV drugs were registered if self-reported at admission and/or in the presence of consistent diagnoses according to the International Classification of Diseases 10th Revision (ICD-10) [21]. 

For the purpose of the present study, we included only AF/AFL patients receiving OAC at discharge, i.e., vitamin K antagonists (VKA) or direct oral anticoagulants (DOAC) according to the current guideline’s recommendations [22] Type of anticoagulant dispensed as well as the prescribed dose was extracted from the Dispensed Drug Registry using the Anatomical Therapeutic Chemical (ATC) classification system (code B01).

**Fig. 1:**
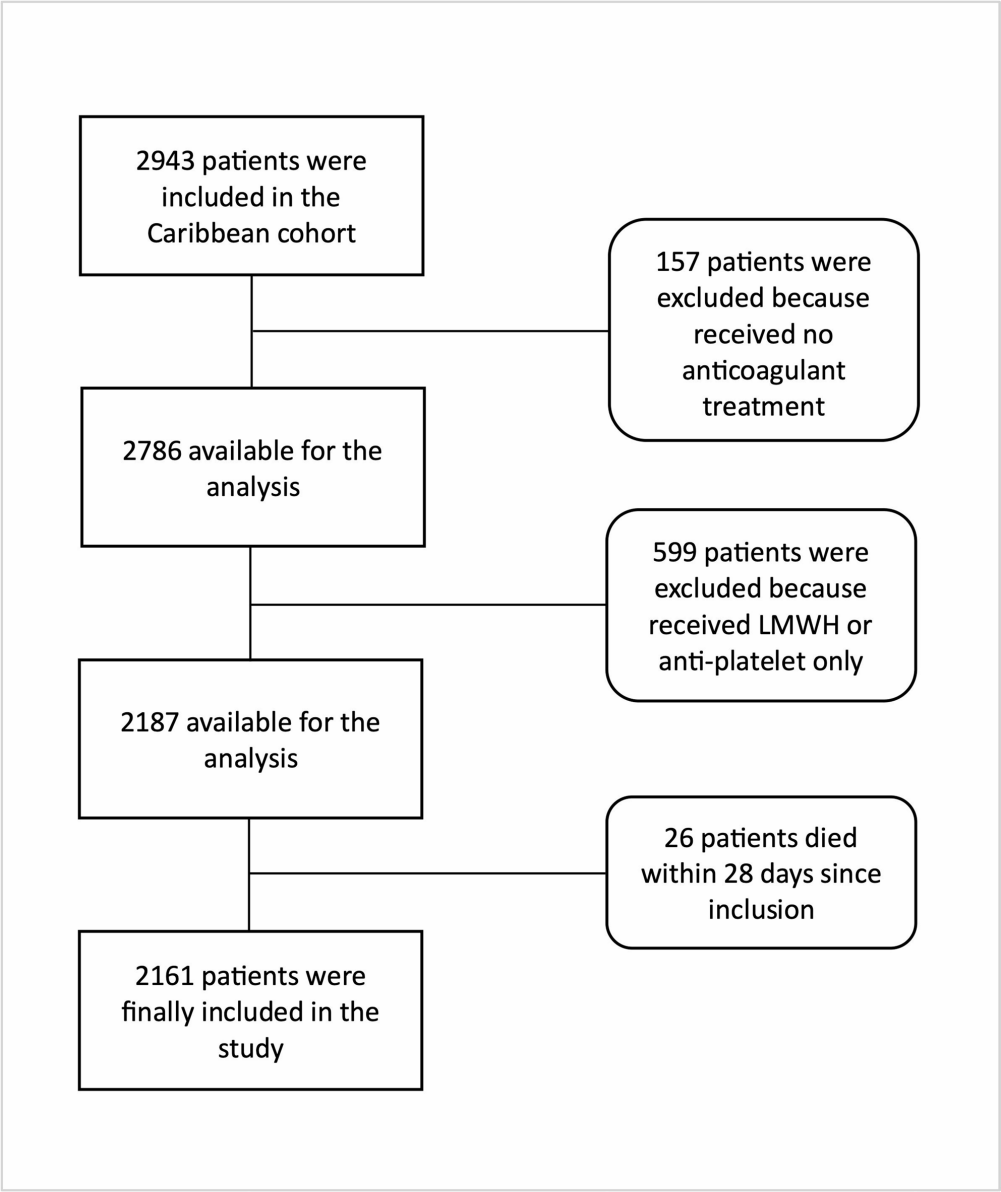
Flowchart summarizing inclusion and exclusion criteria applied in the present study

Patients who died within the first 28 days after discharge, patients who did not receive an OAC prescription or patients only treated with low molecular weight heparin or aspirin were excluded (Fig. 1), leaving 2161 patients to be included in the analysis.

### Hospitalizations for cardiovascular events

We retrieved data on hospitalizations during the first 12 months of follow-up. Diagnoses were retrieved from the National Patient Register (NPR) [23] using appropriate ICD-10 codes. For each hospitalization, we recorded the main diagnosis at discharge and then categorized the diagnoses in 5 groups, summarized in Supplementary Table I. Hospitalization for HF was included in group A; stroke and/or transient ischemic attack (TIA) and systemic embolism (SE) in group B; acute myocardial infarction (AMI) and peripheral artery disease (PAD) in group C; bleeding in group D and all other CV diagnoses in Group E. We recorded all hospitalizations for CVEs with a minimum interval of 28 days between two consecutive admissions. We included bleeding events among the CV hospitalizations because all patients were on anticoagulant therapy, and bleeding is a common and significant cause of hospitalization in cardiovascular settings.

### Outcome definition

All-cause mortality and CV mortality are the outcome in this analysis. Underlying diagnoses for cardiovascular deaths were retrieved from the Swedish Cause of Death Register using appropriate ICD codes, summarized in Supplementary Table II [24]. A priori, we limited the follow-up to the first 12 months after inclusion in the study. This time interval was chosen to obtain complete follow-up data for the whole study cohort and to ensure the least possible variation of baseline characteristics over time.

### Statistical analysis

Continuous and categorical variables were presented as mean and standard deviation (SD), and number and percentage, respectively.

Survival probabilities for all-cause and CV mortality in patients without admission for CVEs versus those with ≥ 1 admissions for CVEs were estimated with the Kaplan-Meier method and visualized through Kaplan-Meier curves. The curves were compared using the log-rank test.

We estimated hazard ratios (HRs) and relative 95% confidence intervals (95% CIs) for all-cause- and CV mortality associated with ≥ 1 hospitalization for each of the CVE groups using Cox regression models. As the same patient could have been admitted more than once, and eventually for different CVEs, each group of CVE diagnosis was considered as a time-dependent covariate. This statistical approach generates a pseudo-cohort, whereby each hospitalization accounts for a “pseudo-patient” of the original cohort [25]. As an example, a patient hospitalized for a given CVE during the follow-up will be handled in the analysis as follows: its observational time will be considered (i) from study inclusion until the date of the first hospitalization; and (ii) from the day following the hospitalization until the end of the observation/death whichever comes first. Similarly, if a patient was hospitalized two times, this patient would contribute thrice to the pseudo-cohort.

The crude Cox model included each one of the five CVE groups and the year of inclusion as covariate. We then built 5 five multivariable Cox models to account for selected risk factors. In addition to age and sex (included in all models), we adjusted by (model 1) hypertension and diabetes; (model 2) comorbidities (i.e., 3 or more chronic diseases); (model 3) prior HF; (model 4) prior stroke or TIA; (model 5) prior bleeding.

Proportional hazard assumptions (PH) for all the six models (i.e., crude model and the five adjusted models) were tested using the Schoenfeld test, with a *p*-value ≤ 0.05 indicating a PH violation. A PH violation, indicating lack of hazards’ proportionality, was detected in all the six models for group A (i.e., HF-related hospitalization), whereas the other covariates (i.e., age, prior bleed, and prior stroke/TIA) had PH violation in select adjusted models only. Thus, after visually exploring residual plots, we split the overall follow-up time into three intervals (0–90 days, 90–180 days, 180–365 days) for these selected groups as this best described trend changes in hazards’ proportionality [25]. Accordingly, HRs for the covariates violating PH assumption were calculated for each of the three follow-up intervals. Schoenfeld test was then repeated to confirm the non-violation of the PH.

A two-sided *p*-value < 0.05 was considered to indicate statistical significance. All analyses were performed using R software 4.0.4.

## Results

### Baseline characteristics of the cohort

Baseline characteristics of the study population are summarized in Table 1. Overall, 185 (8.6%) patients had comorbidities. At baseline, a prior stroke/TIA event was recorded in (%) 404 (18.7) patients, and prior bleeding in 277 (12.8). The most prevalent disease (%) was hypertension (68.7), followed by chronic HF (33.3), and diabetes (15.4).

**Table 1:** Baseline characteristics of the study population

	*N =* 2161
Age (y)	81.91 (5.42)
Male sex, *n* (%)	896 (41.5)
Hypertension, *n* (%)	1486 (68.8)
Diabetes, *n* (%)	333 (15.4)
Prior stroke/TIA*, *n* (%)	404 (18.7)
Prior bleeding, *n* (%)	278 (12.9)
Prior heart failure, *n* (%)	718 (33.2)
Dementia, *n* (%)	117 (5.4)
Cancer, *n* (%)	24 (1.1)
Comorbidities (> 3), *n* (%)	185 (8.6)
Biochemical data (mmol/L)	
Creatinine, mean (SD)	94.36 (39.63)
Hemoglobin, mean (SD)	133.46 (16.09)
Platelet, x1000mm^3^, mean (SD)	228.69 (71.47)
Pharmacological treatment at discharge:	
Anticoagulant treatment	
DOAC*, *n* (%)	797 (36.9)
VKA*, *n* (%)	1364 (63.1)
Concomitant antiplatelet treatment, *n* (%)	53 (2.5)

*Abbreviations*: *TIA *transient ischemic attack, *DOAC *direct oral anti-coagulant, *VKA *vitamin K antagonist

Missing values: Creatinine *n*=6; Hemoglobin *n*= 5; Platelets *n*=12

All patients were treated with OAC: 1364 (63.3%) received VKA whereas the remainders were on apixaban (602; 27.9%), dabigatran (105; 4.8%), and rivaroxaban (88; 4.1%) and 51 (2.4%) patients were on concomitant antiplatelet therapy.

### Hospital admissions according to CVE group

Overall, 391 (18.1%) patients were admitted to the hospital at least once during follow-up and discharged with a main diagnosis consistent with a CVE. As summarized in Table 2, most of the patients were admitted only once, whereas only three (0.1%) were admitted 4 times during the year of follow-up.

**Table 2:** Overall CVEs and total number of CVEs in groups A, B, C, D, E.

Hospitalization number	1	2	3	4	
Group A Heart failure					Group A patients/hospitalizations
1	152	13	3		168/168
2		20	7		27/54
3			6	1	7/21
4				2	2/8
					204/251
Group B Ischemic stroke, TIA, and SE					Group B patients/hospitalizations
1	44	8	2		54/54
2		1			1/2
					55/56
Group C Myocardial infarction					Group C patients/hospitalizations
1	38	11	5		54/54
2		3	1		4/8
					58/62
Group D Bleeding					Group D patients/hospitalizations
1	39	10	1	1	51/51
2		2	2		4/8
					55/59
Group E Other CVEs					Group E patients/hospitalizations
1	42	8			50/50
2		5	1		6/12
					56/62
Total *N *of patients/hospitalizations	315/315	56/112	17/51	3/12	

*Abbreviations:* *CVEs *Cardiovascular events, *SE *systemic embolism. In light-blue patients hospitalized *N* times for the same event

The most common diagnosis for re-admission was HF with a total of 251 hospitalizations. Of these (%), 168 (7.8), 27 (1.2), 7 (0.3), and 2 (0.1) patients were admitted 1, 2, 3, and 4 times for HF, respectively (Table 2). Admissions for other CV causes were relatively less common, as shown in Table 2. Overall, 54 (2.5), and 1 (0.01) patient were hospitalized because of ischemic stroke, TIA, or SE (group B), respectively. Fifty-eight (2.7) patients experienced a total of 62 episodes of the composite outcome of AMI and PAD (group C), while 51 (2.4), and 4 (1.9) patients experienced 1, and 2 episodes of bleeding requiring hospitalization (group D). Finally, 56 (2.6) patients experienced 62 other CVEs (group E).

### CVEs association with all-cause and CV mortality

**Fig. 2:**
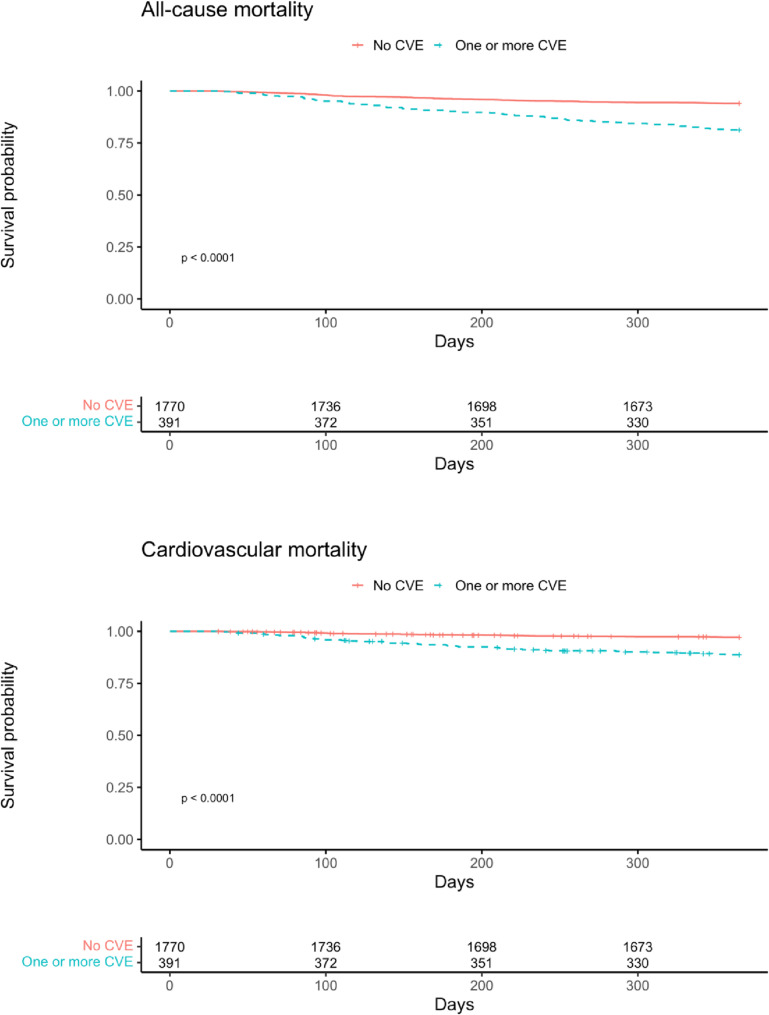
Kaplan - Meier curves for all-cause and cardiovascular mortality. Footnote: CVE cardiovascular event

During a median follow-up of 349 days (range 31 to 365), 178 patients died and out of those 92 deaths were due to CV causes (Fig. 2).

In the HF group, the very high risk of all-cause mortality at 90 days with a HR of 17.43 and 95% CI (9.40–32.34.40.34) was largely reduced with a HR of 3.16 (95% CI 1.82–5.78) between 90 and 180 days, and 3.47 (2.48–4.85) between 180 and 365 days (Table 3). A comparable pattern was observed for CV death (HR and 95% CI) 33.64 (15.97–70.89) at 90 days, 3.73 (1.57–8.85) between 90 and 180 days, and 4.14 (2.73–6.28) after 180 days (Table 4).

**Table 3:** Multivariable Cox models for all-cause mortality before and after adjustment for confounders

Hospitalization cause		Crude	Model 1	Model 2	Model 3	Model 4	Model 5
	*N* event/N reference group	HR 95%CI	HR 95%CI	HR 95%CI	HR 95%CI	HR 95%CI	HR 95%CI
Group A (0–90 days)	13/2218	17.43 (9.40–32.34)	11.5 (6.19–21.36)	11.95 (6.42–22.25)	10.71 (5.73–19.99)	12.17 (6.55–22.61)	12.38 (6.72–22.8)
Group A (90–180 days)	5/2182	3.16 (1.64–6.09)	2.68 (1.41–5.09)	2.75 (1.44–5.24)	2.51 (1.31–4.81)	2.78 (1.46–5.29)	2.75 (1.45–5.22)
Group A (180–365 days)	11/2171	3.47 (2.48–4.85)	3.40 (2.41–4.81)	3.34 (2.36–4.73)	3.18 (2.24–4.51)	3.40 (2.41–4.79)	3.40 (2.42–4.79)
Group B	7/6766	9.64 (4.97–18.70)	8.91 (4.55–17.46)	8.92 (4.55–17.49)	9.08 (4.59–17.97)	8.56 (4.31–17.00)	9.17 (4.65–18.08)
Group C	4/6760	4.62 (2.01–10.63)	4.41 (1.89–10.29)	4.55 (1.97–10.49)	4.12 (1.79–9.50)	4.55 (1.97–10.50)	4.78 (2.06–11.10)
Group D	6/6763	8.33 (4.42–15.71)	6.22 (3.16–12.25)	6.22 (3.17–12.17)	5.74 (2.93–11.25)	6.07 (3.09–11.93)	5.68 (2.86–11.27)
Group E	7/6760	6.86 (3.82–12.33)	6.61 (3.61–12.09)	6.85 (3.74–12.55)	6.73 (3.69–12.28)	6.8 (3.72–12.43)	6.72 (3.69–12.21)

Results are expressed as hazard ratio (95% confidence intervals)

model 1: adjusted for age, sex, hypertension, and diabetes

model 2: adjusted for age, sex, and comorbidities

model 3: adjusted for age, sex, and prior heart failure

model 4 adjusted for age, sex, and prior stroke or TIA

model 5: adjusted for age, sex, and prior bleeding

**Table 4:** Multivariable Cox models for cardiovascular mortality before and after adjustment for confounders

Hospitalization cause		Crude Model	Model 1	Model 2	Model 3	Model 4	Model 5
	*N* event/N reference group	HR 95%CI	HR 95%CI	HR 95%CI	HR 95%CI	HR 95%CI	HR 95%CI
Group A (0–90 days)	11/2218	33.64 (15.97–70.89)	23.86 (10.99–51.79)	22.99 (10.69–49.44)	18.97 (8.82–40.83)	22.88 (10.74–48.74)	22.05 (10.34–46.99)
Group A (90–180 days)	3/2182	3.73 (1.57–8.85)	3.45 (1.46–8.16)	3.39 (1.44–7.99)	2.90 (1.23–6.88)	3.67 (1.47–9.15)	3.40 (1.45–7.96)
Group A (180–365 days)	5/2171	4.14 (2.73–6.28)	3.94 (2.58–6.03)	3.91 (2.55–6.01)	3.57 (2.31–5.51)	3.80 (2.49–5.78)	3.90 (2.56–5.93)
Group B	7/6766	14.73 (7.60–28.58)	12.57 (6.42–24.62)	12.95 (6.62–25.34)	13.96 (7.02–27.75)	9.76 (4.77–19.94)	13.13 (6.63–26.01)
Group C	2/6760	5.70 (1.73–18.75)	5.54 (1.68–18.24)	5.69 (1.73–18.75)	4.92 (1.50–16.09)	6.04 (1.85–19.67)	5.79 (1.76–19.09)
Group D	2/6763	6.41 (1.92–21.44)	4.39 (1.23–15.70)	4.45 (1.25–15.83)	3.98 (1.12–14.19)	3.21 (0.80–12.97)	4.15 (1.10–15.63)
Group E	4/6760	8.98 (4.29–18.78)	9.32 (4.34–20.00)	9.54 (4.45–20.47)	9.15 (4.28–19.52)	9.1 (4.19–19.75)	9.03 (4.23–19.29)

Results are expressed as hazard ratio (95% confidence intervals)

model 1: adjusted for age, sex, hypertension, and diabetes

model 2: adjusted for age, sex, and comorbidities

model 3: adjusted for age, sex, and prior heart failure

model 4 adjusted for age, sex, and prior stroke or TIA

model 5: adjusted for age, sex, and prior bleeding

In patients hospitalized because of ischemic stroke/TIA/SE, the crude HR (95% CI) for all-cause and CV mortality were 17.43 (9.40–32.34) at 365 days (Table 3) and 14.73 (7.60–28.58) (Table 4), respectively.

The crude HR for all-cause mortality was 4.62 (2.01–10.63), and 5.70 (1.73–18.75) for CV mortality (Tables 3 and 4), respectively in patients with AMI and/or PAD.

Those hospitalized for bleeding suffered an increased risk for both all-cause 8.33 (4.42–15.71) and CV mortality 6.41 (1.92–21.44), (Tables 3 and 4, respectively). Similarly, all-cause mortality and CV mortality risks were increased in group E, where all other CV diagnoses were represented (Tables 3 and 4).

Adjustments for additional confounders slightly changed the risk estimates without changing the direction or the validity of the association (Tables 3 and 4), with the exception of the observation that bleeding (group D) was no longer associated with CV mortality (HR 3.21; 95% CI 0.80–12.97) after adjustment by age, sex, and prior stroke/TIA.

### Analysis of the association of risk factors with all-cause and cardiovascular mortality

We then looked at the mortality risk estimates associated with the most common CV risk factors. Increasing age and male sex were associated with higher hazard for all-cause mortality in all of the five Cox models with HR for age ranging from 1.11 to 1.12 and for male sex from 1.55 to 1.59 (Supplementary Table III), in patients admitted once or more than once to the hospital.

Having a history of chronic HF at baseline increased the hazard for all-cause mortality by 55% (HR 1.55; 95% CI 1.14–2.12) whereas the HR for prior bleeding varied during the follow-up, ranging from HR (95% CI) 0.38 (0.12–1.25) to 2.10 (1.25–3.52), at 90, and 365 days, respectively. On the contrary, hypertension, diabetes, prior stroke/TIA, were not associated with all-cause mortality in none of the analyses (Supplementary Table III).

Increasing age, prior HF but not male sex, was associated with higher hazard for CV mortality (Supplementary Table IV). Prior stroke/TIA showed a 4 times greater risk at 180 days (HR 4.28; 95%CI 2.08–8.82), but no association at 90 and 365 days, whereas prior bleeding was not associated with CV mortality (Supplementary Table IV).

## Discussion

In this retrospective study of elderly AF/AFL patients treated with OAC, one fifth of the study population was admitted at least once during 12 months of follow-up to a cardiology department after a hospitalization because of AF/AFL. The impact of hospitalizations for CVEs on survival was noteworthy.

Hospitalization because of HF increased the hazards of all-cause and CV mortality during the first 90 days of observation by 17 and 33 times, respectively. HF and AF often coexist and are linked by a bidirectional relation [26, 27]. AF, atrial myocardial dysfunction and atrial remodeling together constitute the atrial myopathy that makes AF both cause and the consequence of HF [28]. In line with this assumption, 33% of our patients had a history of HF at baseline, and hospitalization because of HF was the most frequent CVE during the follow-up with two patients who experienced up to four episodes of recurrent acute HF. It is reasonable to assume that aging increases this condition because of a longer coexistence of AF and HF. Furthermore, Patel et al. showed how among patients admitted for acute HF, more than half had a history of AF/AFl with a mean age of 74 years (66–82) vs. 63 years (54–71) for those without AF/AFL [29], and the decongestion from the acute HF was blunted in patients with concomitant AF/AFl [29]. Altogether, our and former observations, highlight the higher risk of developing multiple episodes of HF requiring hospitalization in elderly patients with AF/AFl. Considering that the ability to recover after an acute adverse event in elderly is generally low, when frailty co-exist [30], these observations support an earlier, targeted, and maybe more aggressive management of HF in this group of patients. However, HF treatment in elderly patients is challenging due to the presence of co-morbidities, disability, and frailty [31]. As data from clinical trials are scant in population of elderly, this data reinforces the need for clinical trials enrolling elderly and very elderly with HF and AF.

Despite all the patients in our cohort being treated with OAC as per guidelines recommendation, ischemic stroke/TIA, and SE (group B) were relatively common and associated with a remarkable risk for all-cause mortality, and even a higher risk for CV mortality. In a meta-analysis of the four pivotal trials comparing DOAC vs. VKA in AF, the pooled incidence of the composite outcome (stroke and SE) was about 3.4. and 4.2% in the subgroup of patients being 75 years or older, respectively [32]. As expected, we found similar estimates (about 3.9%) when limiting the observation to the first hospitalization for CVE; however, this estimate increased till 5.5% when we considered multiple CVEs. This is of interest because studies on AF management and stroke prevention often limit the observation at the first CVE. While this approach is pivotal in analyzing the safety and the effectiveness of the OAC treatment in the general population, it may underestimate the clinical complexity of elderly patients with AF. However, the present study demonstrates that although reduced, the weight of stroke/TIA or systemic embolism is still burdensome. This concept has already been highlighted in the GLORIA-AF Registry [33] Of relevance, we have shown in this population that switching from a non-guideline recommended treatment to OAC treatment as per guidelines recommendation reduced the occurrence of adverse thrombotic events in elderly AF patients [34]. We believe it is reasonable to consider, especially in future studies on the subject, the impact that multiple hospitalizations for CVEs may have on the incidence of stroke/TIA and systemic embolism. Indeed, multiple hospitalizations can lead to discontinuation of medications, or they may require the prescription of additional medications that can alter the functionality of several drugs including anticoagulants, especially in elderly [35, 36], making this an important residual confounder in observational studies not taking hospitalizations into account.

Our data extend previous knowledge on the safety of the OACs in this patients group, given the low incidence of hospitalization because of bleeding events. Compared with clinical trial results, we found a lower incidence of bleeding (2.7% vs. 12.5%) even considering multiple bleeding events [32].

A meta-analysis from 2011 showed how new-onset AF following an AMI increased mortality, suggesting that AF should be considered as a severe event during AMI [37]. Conversely, the higher risk of AMI in AF patients has been well documented [38]. Similar to HF, AF and AMI are also related by a two-way link, due the common risk factors that AF and coronary artery disease both share [39]. We confirmed the association between AMI and AF, and we have also shown that considering multiple admissions for different CVE increases the magnitude of the mortality risk for AMI.

Finally, the HR for all-cause mortality for group E of CVEs were approximately 6 in all the Cox’s models while the same figure was around 9 for cardiovascular mortality. This group well displays the burden carried by heart valve diseases or pericardial/myocardial inflammatory or infectious heart diseases or venous thromboembolism. In the last decades the advances in heart valve diseases management or in venous thromboembolism treatment seemed to have improved the quality of life in elderly patients [40, 41]. However, further studies with tailored approaches are needed to promote survival among these fragile patients.

Among risk factors associated with CVEs, most of them were not modifiable like age, sex, prior bleeding, prior HF, or prior thrombotic event. All of them have already been found associated with higher risk for CVEs in previous studies [42–44]. While we can confirm the association between some of the aforementioned risk factors (i.e., age, sex, prior HF, prior stroke/TIA), the magnitude of these associations was generally low. Therefore, the use of these risk factors for prognostic or predictive purposes should be considered with extreme caution.

Our study has strengths and limitations. The strength resides in the national coverage of registries and the granularity of the clinical data, which allow us to follow during time the clinical history of a patient including dispensed medicines. We provide a clear representation of the real-life clinical history of unselected consecutive elderly patients with AF on treatment with OAC and highlight the importance of considering admissions for CVEs to better understand the complexity and to stratify the mortality risk of these specific patients.

However, the results of our study need to be interpreted with caution considering several limitations. This is a Swedish single center study, with a limited generalizability to other clinical settings. There was no standardization in the anticoagulant prescription, and the choice was left to the attending clinician’s judgment. This did not allow a more in-depth analysis according to the type of anticoagulant used (VKA vs. OAC) chosen. Moreover, none was treated with edoxaban. Hence, these estimates may not apply to elderly AF patients on edoxaban. Also, management of the different CVEs was not standardized. For this reason, we cannot exclude that different approaches may have produced different results in terms of recurrent or new CVEs during the follow-up. Eventually, our analysis strategy does not provide information on possible causality between two or more consecutive CVEs.

The novelty of our study lies in providing a reliable estimation of how repeated hospitalizations for various cardiovascular causes can significantly influence survival probabilities in elderly and very elderly patients. Our use of a time-varying approach yields different and more comprehensive estimates compared to traditional non-time-varying methods (discussed above), which only consider the first event. While useful, these methods do not fully capture the dynamic clinical course of these patients. In contrast, our methodology reflects the evolving health status of patients, offering a more accurate understanding of the impact of recurrent hospitalizations on survival.

In light of the relatively high frequency of hospitalizations among elderly and very elderly patients with AF, and considering the associated reduction in life expectancy, it would be reasonable for clinicians to shift some focus toward optimizing palliative and end-of-life care strategies. Addressing this aspect could help reduce the burden linked to recurrent hospitalizations, improve overall quality of life, and ensure that patient care aligns with their clinical priorities and preferences. This approach underscores the importance of comprehensive management that not only aims to improve survival but also emphasizes patient comfort and dignity during advanced stages of illness.

In conclusion, the occurrence of one or more hospitalization because of a CVE in elderly AF patients on treatment with OAC negatively impact survival. Despite OAC, residual risk of stroke/TIA is noteworthy and associated with high cardiovascular mortality. Acute HF is the most prevalent CVE. Beyond anticoagulant treatment, tailored approaches are utterly needed to improve survival and quality of life in elderly AF patients with atrial fibrillation. We believe that these findings may provide a useful guide for future dedicated studies looking at improving the prognosis and extending the healthy span of elderly AF patients, even if the importance of considering multiple hospitalizations to improve mortality risk scores remains to be investigated.

## Supplementary Information

Supplementary Material 1.

## Data Availability

The data that support the findings of this study are available from the corresponding author, B.G., upon reasonable request.
