# Supplementary material for: Hospitalizations for cardiovascular events and risk for all cause and cardiovascular mortality in elderly patients with atrial fibrillation treated with oral anticoagulants: beyond preventing thromboembolism
Source: BMC Geriatr. 2025 Dec 9;25:1007. doi: 10.1186/s12877-025-06733-8 (PMC12687549; doi:10.1186/s12877-025-06733-8)
Supplement: Supplementary file 1 — Supplementary Material 1. [file 12877_2025_6733_MOESM1_ESM.docx]

**SUPPLEMENTARY MATERIAL**

### **Hospitalizations for cardiovascular events and risk for all cause and cardiovascular mortality in elderly patients with atrial fibrillation treated with oral anticoagulants: beyond preventing thromboembolism**

Matteo Candeloro^1,2^, Qiaosen Chen^2^, Hanne Ehrlinder^3,4^, Bruna Gigante^2,4^

^1^ Department of Innovative Technologies in Medicine and Dentistry, “G. D’Annunzio” University, Chieti, Italy

^2^ Division of Cardiology, Department of Medicine Solna, Karolinska Institutet, Solnavägen 30, 171 64 Stockholm, Sweden.

^3^ Department of Clinical Sciences, Danderyd Hospital, Division of Cardiovascular Medicine, Karolinska Institutet, Stockholm, Sweden

^4^ Department of Cardiology, Danderyd University Hospital, Entrevägen 2, 182 88 Stockholm, Sweden.

**Corresponding author:** Bruna Gigante, Division of Cardiology, Department of Medicine Solna, Karolinska Institutet, Solnavägen 30, 171 64 Stockholm, Sweden. Tel.: 0046 8 52487082

E-mail address: bruna.gigante@ki.se

**Supplementary Table I**. Groups of cardiovascular events according to ICD codes

| CVE | ICD codes | |
| --- | --- | --- |
| Group A  Heart Failure | I500, I501, I509 |  |
| Group B  Ischemic stroke, TIA, SE | G453, G459, I633, I634, I635, I639, I693, I694 |  |
| Group C  Acute myocardial infarction, peripheral artery disease | I200, I209, I210, I212, I214, I219, I250, I251, I252, I259, I702, I702C, I702X, I743 |  |
| Group D  Bleeding | D500, D629, D683, I610, I612, I618, I619, I620, I690B, K250, K260, K625, K921, K922, N950, N950X, R040, R319, R589, S065, S0650, S0660 T140A, T810 |  |
| Group E  Other disease | D509, I269, I270, I313, I330, I340, I342, I350, I351, I369, I409, I429, I460, I469, I710, I714, I951, I952, I959, Z432 |  |

CVE: cardiovascular events

**Supplementary Table II**. Cause and number of cardiovascular death according to ICD codes

| Cause of Death | N | ICD codes |
| --- | --- | --- |
| Cardiac/vascular | 39 (1.80%) | I059, I080, I340, I350, I408, I461, I48, I482, I489, I516, I519, I723 |
| Thrombo/cardioembolism/atherothrombosis | 30 (1.39%) | I209, I219, I251, I258, I259, I272, I635, I639, I64, I698, I709, I739 |
| Heart Failure | 21 (0.97%) | I501, I509 |
| Bleeding | 2 (0.09%) | I609, I619 |

**Supplementary Table III.** Multivariable Cox models for all-cause mortality before and after adjustment for confounders.

| Hospitalization cause | Crude | Model 1 | Model 2 | Model 3 | Model 4 | Model 5 |
| --- | --- | --- | --- | --- | --- | --- |
|  | HR 95%CI | HR 95%CI | HR 95%CI | HR 95%CI | HR 95%CI | HR 95%CI |
| Group A  (0-90 days) | 17.43 (9.40 - 32.34) | 11.5 (6.19 - 21.36) | 11.95 (6.42 - 22.25) | 10.71 (5.73 - 19.99) | 12.17 (6.55 - 22.61) | 12.38 (6.72 - 22.8) |
| Group A  (90-180 days) | 3.16 (1.64 - 6.09) | 2.68 (1.41 - 5.09) | 2.75 (1.44 - 5.24) | 2.51 (1.31 - 4.81) | 2.78 (1.46 - 5.29) | 2.75 (1.45 - 5.22) |
| Group A  (180-365 days) | 3.47 (2.48 - 4.85) | 3.40 (2.41 - 4.81) | 3.34 (2.36 - 4.73) | 3.18 (2.24 - 4.51) | 3.40 (2.41 - 4.79) | 3.40 (2.42 - 4.79) |
| Group B | 9.64 (4.97 - 18.70) | 8.91 (4.55 - 17.46) | 8.92 (4.55 - 17.49) | 9.08 (4.59 - 17.97) | 8.56 (4.31 - 17.00) | 9.17 (4.65 - 18.08) |
| Group C | 4.62 (2.01 - 10.63) | 4.41 (1.89 - 10.29) | 4.55 (1.97 - 10.49) | 4.12 (1.79 - 9.50) | 4.55 (1.97 - 10.50) | 4.78 (2.06 - 11.10) |
| Group D | 8.33 (4.42 - 15.71) | 6.22 (3.16 - 12.25) | 6.22 (3.17 - 12.17) | 5.74 (2.93 - 11.25) | 6.07 (3.09 - 11.93) | 5.68 (2.86 - 11.27) |
| Group E | 6.86 (3.82 - 12.33) | 6.61 (3.61 - 12.09) | 6.85 (3.74 - 12.55) | 6.73 (3.69 - 12.28) | 6.80 (3.72 - 12.43) | 6.72 (3.69 - 12.21) |
| Age | - | 1.12 (1.09 - 1.15) | 1.12 (1.09 - 1.15) | 1.11 (1.08 - 1.14) | 1.12 (1.09 - 1.15) | 1.12 (1.09 - 1.15) |
| Sex, male | - | 1.57 (1.16 - 2.14) | 1.57 (1.16 - 2.13) | 1.55 (1.14 - 2.11) | 1.59 (1.17 - 2.16) | 1.56 (1.15 - 2.13) |
| Prior hypertension | - | 1.14 (0.81 - 1.60) | - | - | - | - |
| Prior diabetes | - | 1.41 (0.98 - 2.04) | - | - | - | - |
| Comorbidities (≥3) | - | - | 1.31 (0.83 - 2.06) | - | - | - |
| Prior HF | - | - | - | 1.55 (1.14 - 2.12) | - | - |
| Prior stroke/TIA | - | - | - | - | 1.09 (0.77 - 1.55) | - |
| Prior bleed | - | - | - | - | - | - |
| Prior bleed (0-90 days) | - | - | - | - | - | 0.38 (0.12 - 1.25) |
| Prior bleed (90-180 days) | - | - | - | - | - | 0.81 (0.40 - 1.67) |
| Prior bleed (180-365 days) | - | - | - | - | - | 2.10 (1.25 - 3.52) |

Supplementary table 4. Multivariable Cox models for cardiovascular mortality before and after adjustment for confounders.

| Hospitalization cause | Crude | Model 1 | Model 2 | Model 3 | Model 4 | Model 5 |
| --- | --- | --- | --- | --- | --- | --- |
|  | HR 95%CI | HR 95%CI | HR 95%CI | HR 95%CI | HR 95%CI | HR 95%CI |
| Group A  (0-90 days) | 33.64 (15.97 - 70.89) | 23.86 (10.99 - 51.79) | 22.99 (10.69 - 49.44) | 18.97 (8.82 - 40.83) | 22.88 (10.74 - 48.74) | 22.05 (10.34 - 46.99) |
| Group A  (90-180 days) | 3.73 (1.57 - 8.85) | 3.45 (1.46 - 8.16) | 3.39 (1.44 - 7.99) | 2.90 (1.23 - 6.88) | 3.67 (1.47 - 9.15) | 3.40 (1.45 - 7.96) |
| Group A  (180-365 days) | 4.14 (2.73 - 6.28) | 3.94 (2.58 - 6.03) | 3.91 (2.55 - 6.01) | 3.57 (2.31 - 5.51) | 3.80 (2.49 - 5.78) | 3.90 (2.56 - 5.93) |
| Group B | 14.73 (7.60 - 28.58) | 12.57 (6.42 - 24.62) | 12.95 (6.62 - 25.34) | 13.96 (7.02 - 27.75) | 9.76 (4.77 - 19.94) | 13.13 (6.63 - 26.01) |
| Group C | 5.70 (1.73 - 18.75) | 5.54 (1.68 - 18.24) | 5.69 (1.73 - 18.75) | 4.92 (1.50 - 16.09) | 6.04 (1.85 - 19.67) | 5.79 (1.76 - 19.09) |
| Group D | 6.41 (1.92 - 21.44) | 4.39 (1.23 - 15.70) | 4.45 (1.25 - 15.83) | 3.98 (1.12 - 14.19) | 3.21 (0.80 - 12.97) | 4.15 (1.10 - 15.63) |
| Group E | 8.98 (4.29 - 18.78) | 9.32 (4.34 - 20.00) | 9.54 (4.45 - 20.47) | 9.15 (4.28 - 19.52) | 9.10 (4.19 - 19.75) | 9.03 (4.23 - 19.29) |
| Age | - | 1.12 (1.08 - 1.16) | 1.12 (1.08 - 1.17) | 1.11 (1.07 - 1.15) | - | - |
| Age (0-90 days) | - | - | - | - | 1.18 (1.10 - 1.27) | 1.18 (1.10 - 1.27) |
| Age (90-180 days) | - | - | - | - | 1.13 (1.07 - 1.2) | 1.16 (1.10 - 1.23) |
| Age (180-365 days) | - | - | - | - | 1.06 (0.99 - 1.13) | 1.06 (0.99 - 1.12) |
| Sex | - | 1.09 (0.7 - 1.7) | 1.05 (0.68 - 1.65) | 1.04 (0.66 - 1.62) | 1.04 (0.67 - 1.63) | 1.05 (0.67 - 1.65) |
| Prior hypertension | - | 1.24 (0.76 - 2.02) | - | - | - | - |
| Prior diabetes | - | 1.00 (0.56 - 1.77) | - | - | - | - |
| Comorbidities (≥3) | - | - | 1.45 (0.78 - 2.7) | - | - | - |
| Prior HF | - | - | - | 2.19 (1.4 - 3.42) | - | - |
| Prior stroke/TIA (0-90 days) | - | - | - | - | 0.21 (0.05 - 0.93) | - |
| Prior stroke/TIA (90-180 days) | - | - | - | - | 4.28 (2.08 - 8.82) | - |
| Prior stroke/TIA (180-365 days) | - | - | - | - | 1.82 (0.88 - 3.75) | - |
| Prior bleed (0-90 days) | - | - | - | - | - | 0.17 (0.02 - 1.29) |
| Prior bleed (90-180 days) | - | - | - | - | - | 0.88 (0.33 - 2.35) |
| Prior bleed (180-365 days) | - | - | - | - | - | 2.1 (0.96 - 4.58) |
